# Winter Waterbird Community Composition and Use at Created Wetlands in West Virginia, USA

**DOI:** 10.1155/2017/1730130

**Published:** 2017-03-12

**Authors:** Hannah L. Clipp, Michael L. Peters, James T. Anderson

**Affiliations:** ^1^School of Natural Resources, West Virginia University, P.O. Box 6125, Morgantown, WV 26505, USA; ^2^West Virginia Division of Natural Resources, P.O. Box 99, Farmington, WV 26571, USA

## Abstract

Information on nonbreeding waterbirds using created wetlands in the Central Appalachian region of the United States is limited. We compared waterbird communities of two managed wetlands, created in 2013 and 2001, in West Virginia. We observed 27 species of waterbirds. Species richness and diversity were generally similar between the wetlands, but species composition and use differed.* Branta canadensis* (Canada Geese),* Anas strepera* (Gadwall),* Bucephala albeola* (Buffleheads),* Aythya affinis* (Lesser Scaup), and* Aythya collaris* (Ring-Necked Ducks) used the older wetland most frequently. Disparities in species use were the highest in March. The older wetland differed from the younger in supporting species such as diving ducks, possibly due to differences in size, vegetation, water depth, and microtopography. However, the ability to provide habitat for waterbirds during the winter was determined to be comparable between wetlands, despite their age difference.

## 1. Introduction

Wetlands provide an assortment of ecosystem services, such as flood control, nutrient cycling, water filtration, and pollution removal [1, 2]. They can improve water quality, control shoreline erosion, provide natural products, and contribute to the economics of fishing, hunting, agriculture, and recreation [3]. In addition, wetlands are complex ecosystems that provide habitat for a diversity of animals, including insects, mollusks, fish, amphibians, mammals, and birds [4]. Though wetlands comprise a small percentage of the nation's total land area (~5.5%), they harbor a disproportionately high number of unique plants and animals [5]. In the United States, at least one-third of threatened and endangered species lives in or depends on wetlands [6].

Wetlands within the migratory and wintering ranges of waterbird species are critical to conserve and sustain their populations. Waterbirds use coastal and inland wetlands as stopover sites during migration and as habitat to rest, feed, or overwinter [7, 8]. For example, vegetated playa wetlands on the Southern High Plains of Texas can support thousands of waterbirds between November and January [7]. Tens of thousands of waterbirds use wetlands in the San Joaquin Valley of California in January and February [9]. Wetlands can also be important in conserving endangered and threatened bird species, such as* Rallus crepitans* (Gmelin) (Clapper Rails) and* Ammospiza maritimus* (Wilson) (Seaside Sparrows) [10]. The loss of wetlands may explain the declining populations of certain waterbirds [11].

Despite their many benefits, wetlands tend to conflict with competing land and resource development interests. Over the past 2 centuries, many wetlands have been destroyed, converted for agricultural purposes, developed, or manipulated for other human uses. From the 1780s to the 1980s, the conterminous United States lost 53% of its original wetlands [12]. Due to the severe historic loss of wetlands, the United States adopted a national policy of “no net loss of wetlands.” Destruction or degradation of wetlands now requires permits and usually entails either on-site mitigation or mitigation of wetlands of the same size or larger and similar functions in another location. Due to the “no net loss” policy, thousands of hectares of wetlands have been created or restored in compensation for wetland destruction and disturbance due to human activities. For instance, 198,230 ha of former upland were converted to wetlands and an estimated 83,890 ha of freshwater ponds were created from 2004 to 2009 [13].

Several studies have focused on wetland functions and communities within the Central Appalachian region. In West Virginia, Gingerich and Anderson [14], Gingerich et al. [15], and Balcombe et al. [16] examined litter decomposition and plant communities, respectively, in mitigated and reference wetlands. Francl et al. [17] surveyed small mammal communities at wetlands in West Virginia and Maryland. Strain et al. [18] investigated the diet composition and selection of prey by* Notophthalmus viridescens viridescens* (Rafinesque) (Red-spotted Newt) in created and natural wetlands in the Central Appalachians to assess functional equivalency between the wetlands. In addition, Balcombe et al. [19–21] compared aquatic macroinvertebrate, anuran, and breeding season avian assemblages in mitigation and reference wetlands. Although Balcombe et al. [19, 20] found that the mitigation wetlands in their study provided quality habitat for wildlife, they do not all match the function and structure of natural or reference wetlands [22–26]. Thus, it is critical to assess and monitor how created and mitigated wetlands function in offering the same ecological services as natural or reference wetlands. The aforementioned research has been valuable in evaluating the success of mitigation wetlands in supporting wildlife taxa, but there are few studies that specifically focus on waterbird use of created wetlands in the Central Appalachians and even fewer that focus on winter or nonbreeding waterbird communities.

In the summer of 2013, the West Virginia Division of Natural Resources (WVDNR) partnered with West Virginia University and AllStar Ecology LLC to create a mitigated wetland in the Pleasant Creek Wildlife Management Area (WMA), located in north-central West Virginia. The created wetland (hereafter referred to as PC2013) is one of few wetlands in West Virginia managed specifically for the benefit of migratory and wintering waterbirds (e.g., food-producing vegetation was planted and water levels are manipulated). The WVDNR's primary goal was to develop the wetland for waterfowl use and for both consumptive and nonconsumptive waterfowl recreation. It is generally assumed that created wetlands will provide the same ecological services as a natural wetland, but it is not guaranteed, and wetland age may be a confounding factor [24]. Therefore, the purpose of this study was to assess and compare the winter waterbird communities of the recently created wetland and an adjacent older wetland created in 2001 (hereafter referred to as PC2001) in the Pleasant Creek WMA. Our objectives were to (1) perform weekly waterbird surveys at PC2013 and PC2001 from November to March of 2013-2014 and 2014-2015 to determine nonbreeding waterbird use in this region; (2) compare annual and monthly waterbird species richness, diversity, composition, and use at the 2 differently aged wetlands; (3) examine trends in waterbird use during the study period; and (4) determine whether the recently created PC2013 was providing comparable winter waterbird habitat to an older wetland.

## 2. Materials and Methods

### 2.1. Field-Site Description

**Figure 1:**
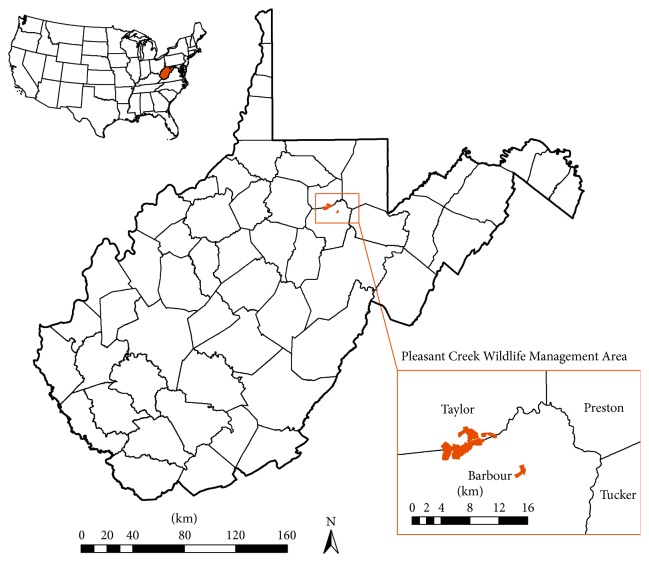
Location of Pleasant Creek Wildlife Management Area, WV. The shaded area at the border of Taylor and Barbour Counties represents the Pleasant Creek Wildlife Management Area, which is managed by the West Virginia Division of Natural Resources.

Our study took place in the Pleasant Creek WMA, located in the Tygart Valley watershed of north-central West Virginia, USA (Figure 1). The 2 study sites included the newly created wetland and an established wetland, which are found in the eastern portion of the Pleasant Creek WMA, near the junction of Taylor and Barbour Counties. A portion of the Pleasant Creek WMA is part of the U.S. Army Corps of Engineers (USACE) Tygart Lake flood control project. The USACE owns land up to the elevation of 362.7 m on the WMA, and the remainder is owned by WVDNR; the entire area is managed by the WVDNR Wildlife Resources Section. The Pleasant Creek WMA consists of mixed hardwood forest and wetland area, totaling 1,226 ha, with moderately steep slopes rising to 488 m in elevation. The area is primarily used for hunting, viewing wildlife, and recreational fishing.

The Pleasant Creek WMA is located within the Appalachian Plateau physiographic province. The underlying rock in this region is sedimentary, and streams tend to be dendritic. The regional climate is generally considered to be humid continental, with humid summers and cool to cold winters. The average precipitation for this region falls between 381 and 442 cm, with temperatures ranging from −3.3 to 5.0°C in January and 19.4 to 24.4°C in July. Because of the area's valley topography, dense fogs are a common occurrence. Cloudy skies are also frequent due to the damming of moisture from the Appalachian Mountains.

Prior to creation, PC2013 had been a maintained field dominated by* Phalaris arundinacea* L. (Reed Canary Grass), an invasive species with minimal value as waterbird and wildlife habitat. In conjunction with West Virginia University, AllStar Ecology LLC, and the Tygart Valley Conservation District, the WVDNR oversaw the creation of the 2.96-ha wetland. Funded by wetland mitigation money received by the WVDNR, the restoration project commenced in June 2013 and construction was mostly completed by August 2013. AllStar Ecology LLC developed the site plans, and the Tygart Valley Conservation District conducted the earthwork. Drainage tiles were removed, deep pockets were excavated, berms were created, and water control devices were installed. The Reed Canary Grass was controlled and a more natural hydrology was restored, creating conditions more suited to native wetland vegetation.* Trifolium repens* L. (Will Ladino Clover),* Lolium perenne* L. (Perennial Rye), and* Echinochloa esculenta* (A. Braun) H. Scholz (Japanese Millet) were planted on the berm and in the wetland after construction during the 2013 growing season. A native wetland seed mix was sowed during the 2014 growing season.

**Figure 2:**
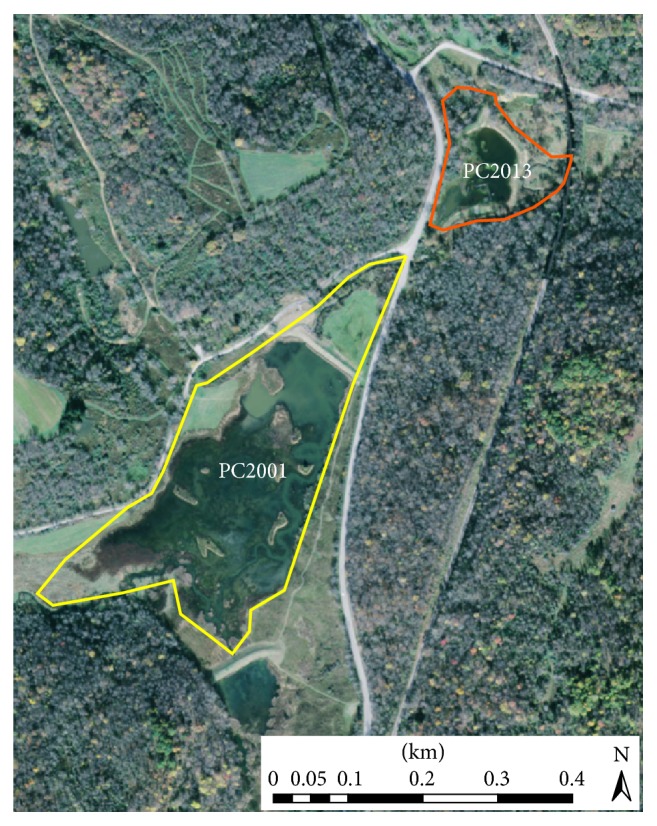
Aerial view of the wetland created in 2001 (PC2001) and the wetland created in 2013 (PC2013). PC2001 is outlined in yellow, while PC2013 is outlined in orange.

PC2013 is mostly bordered by forest, with the northern portion partly under tree and shrub cover (Figure 2). A small stream (Pleasant Creek) runs along the eastern and southern boundaries of the wetland, adding to habitat complexity. The water depth of PC2013 is relatively shallow, averaging 0.45–0.61 m, with a maximum of 1.4 m. The depth can be manipulated by the WVDNR to meet waterbird needs or other objectives. In comparison, PC2001 is larger in area (13.78 ha) and contains 7 islets. Pleasant Creek runs through the wetland, entering from the southern end and exiting from the northern portion (headed downstream to PC2013). A combination of forest, small fields (<0.4 ha), and roads border the wetland (e.g., Pleasant Creek Road runs along the western edge while Route 119 flanks the eastern edge). The water of PC2001 is generally deeper, with a maximum depth over 1.83 m in some areas. According to land cover data from the WVDNR's 2015 Terrestrial Habitat Map, both wetlands are characterized predominantly by open water and small stream riparian habitat, with minor developed sections from bordering roads [27]. Based on a 25 m buffer around the edge of each wetland, PC2001 has 5.29 ha of core habitat (39.2%), while PC2013 has 0.16 ha of core habitat (5.3%). Together, the 2 wetlands total an area of roughly 16.74 ha.

### 2.2. Waterbird Surveys

We conducted weekly surveys from November to March in 2013-2014 and 2014-2015. Each wetland was surveyed 2 or 4 times per week by 1-2 trained observers. We conducted half of the surveys during morning hours, beginning within 30 minutes of sunrise, and half of the surveys during the evening, ending within 30 minutes of sunset, as dawn and dusk are primary waterbird foraging hours [28, 29]. Surveys were conducted on foot and by vehicle, and birds were identified from a distance with binoculars and a spotting scope to avoid disturbance. Waterbirds were considered waterfowl, seabirds, shorebirds, wading birds, and* Megaceryle alcyon* L. (Belted Kingfishers) [30]. Half of the morning and evening surveys proceeded starting at PC2001 and half starting at PC2013. The amount of time spent at each wetland was standardized at 30 minutes. Surveys at PC2013 were conducted by walking along the water's eastern edge and at PC2001 by walking or driving along the northern and western boundaries of the wetland, periodically stopping at locations from which a large portion of the wetland could be observed. Each waterbird was identified to species and sex when possible. To avoid pseudoreplication or double-counts, we systematically and sequentially surveyed sections of the wetland that did not overlap, making note of the species, sex, and number of waterbirds that flushed or swam from one section to another.

The study was focused on waterbirds that were actively using the wetlands; therefore, we recorded only waterbirds observed in the wetland or within 10 m of the wetland's boundary. The small size and accessibility of the wetlands allowed for total counts of waterbirds. Birds that flew over the wetlands but were not foraging or actively using the wetland were not included in the analyses. Additional data collected included the Julian date, times that the survey started and ended, air temperature, and percent ice cover (i.e., the percentage of wetland area covered by ice). Ice cover was tested as a possible explanatory factor for no waterbird detection during the winter surveys.

### 2.3. Comparing Waterbird Communities

To analyze the data and assess the ability of PC2013 to provide waterbird habitat in comparison to the older PC2001, we compared overall, annual, and monthly waterbird species richness, diversity, composition, and use (dependent variables) at each wetland (independent variable). Because we could not confidently identify when individual waterbirds used the wetland multiple days, species use was quantified as the highest species count of each week's surveys (e.g., if 2* Anas platyrhynchos* L. [Mallards] were seen during one survey and 5 were seen during a second survey within the same week, the quantity of 5 Mallards would be used). Species richness was compared using single-factor analysis of variance (ANOVA) with an alpha level of 0.05. For significant results, we further divided species richness by the area of the wetland (ha) and performed ANOVA analyses again to compensate for size differences. Species diversity was calculated using the Shannon-Wiener Diversity Index and compared using single-factor ANOVA. Percent species composition was determined by dividing the total number of an individual species by the total amount of individuals detected within the time period (e.g., month, year), and then it was compared using Schoener's Index to determine percent overlap of communities and *G*-tests. Species use was designated as waterbirds per ha and then compared using single-factor ANOVA.

Variation between wetlands was compared using overall metrics that were combined from the entire survey period. The means from the ANOVA tests were derived from monthly count data (e.g., overall species diversity for PC2001 was calculated using the monthly diversity values from November to March 2013-2014 and 2014-2015 data) and weekly high species counts (e.g., for comparing species use). Variation between wetlands by year was compared using annual metrics. Thus, the means from the ANOVA tests were derived from values from the monthly count data (e.g., annual species diversity for PC2001 in 2013-2014 was calculated using the diversity values from November 2013, December 2013, January 2014, February 2014, and March 2014) and weekly high species counts. Variation between wetlands by month was compared using average monthly metrics that were derived from the weekly surveys conducted within that month. *G*-tests were used to compare the species composition of both wetlands for each month in the 2 survey periods. To determine differences in monthly species use, ANOVA was used to compare average weekly high species counts. Each statistical test was then run through the sequential Bonferroni approach to minimize type I errors [31].

In addition, we examined trends in waterbird use by creating use curves (waterbirds/ha plotted against time) for waterbird species that comprised at least 2.0% of the species composition at either wetland during the 2 years of surveys. Monthly use was calculated by averaging the weekly high species counts and dividing by the number of hectares encompassed by the corresponding wetland. Average waterbird use per ha was plotted for each month in the study. The use curves allow us to determine when waterbirds are using the 2 wetlands and visualize in which months use is similar or diverges.

## 3. Results

### 3.1. Survey Data

During November to March 2013-2014, we conducted 127 surveys and observed 1,831 waterbirds (*n* = 1,749 at PC2001, *n* = 82 at PC2013) belonging to 23 species (*n* = 23 at PC2001, *n* = 7 at PC2013). The average temperature and percent ice cover were 1.8°C and 54%, respectively. In the following year (November to March 2014-2015), we conducted 121 surveys and observed 1,509 waterbirds (*n* = 1,309 at PC2001, *n* = 200 at PC2013) belonging to 24 species (*n* = 24 at PC2001, *n* = 10 at PC2013). The average temperature was similar to the previous year (1.2°C), while average percent ice cover was slightly lower (49%).

Combining the 2 years, a grand total of 248 surveys (124 surveys at each wetland) were conducted from November 2013 to March 2015, and 3,340 waterbirds (*n* = 3,058 at PC2001, *n* = 282 at PC2013) belonging to 27 species (*n* = 27 at PC2001, *n* = 11 at PC2013) were observed. Common disturbances at the 2 wetlands during both years included the presence of hunters and noise from a nearby shooting range and railroad trestle. Average temperatures during surveys across the 2 years ranged from −3.7°C to 4.6°C, and average ice cover was approximately 50%. The proportion of surveys in which no waterbirds were observed was similar between years (40%).

### 3.2. Variation between Wetlands

Overall average species richness initially appeared higher at PC2001, but further analyses to compensate for the effects of wetland size revealed no significant difference (*P* = 0.108). Species diversity did not significantly differ either (Table 1). There was a 33.0% overlap in species composition between the 2 wetlands. PC2001 had a significantly higher percent composition of* Bucephala albeola* L. (Bufflehead; PC2001 = 10.73%, PC2013 = 0.71%, *G*_1_ = 10.5, *P* < 0.005),* Anas strepera* L. (Gadwall; PC2001 = 7.03%, PC2013 = 0.0%, *G*_1_ = 9.7, *P* < 0.005),* Aythya affinis* (Eyton) (Lesser Scaup; PC2001 = 8.24%, PC2013 = 0.0%, *G*_1_ = 11.4, *P* < 0.001), and* Aythya collaris* (Donovan) (Ring-Necked Duck; PC2001 = 17.5%, PC2013 = 0.0%, *G*_1_ = 24.3, *P* < 0.001), while PC2013 had a greater percent composition of Mallard (PC2001 = 4.74%, PC2013 = 23.40%, *G*_1_ = 13.5, *P* < 0.001) and* Aix sponsa* L. (Wood Duck; PC2001 = 1.86%, PC2013 = 33.33%, *G*_1_ = 34.2, *P* < 0.001). The percent composition of the other 21 species was similar between the 2 wetlands (*P* > 0.05).

Average total species use over the course of the 2 survey periods was higher at PC2001. Three of the 27 species had significant differences in use. Buffleheads,* Branta canadensis* L. (Canada Goose), and Gadwall all had higher use values at PC2001 (Table 1). Furthermore, the proportion of surveys in which no waterbirds were observed was greater at PC2013 (PC2001: 0.23, PC2013: 0.58, *P* < 0.05), but there was no difference between the average percent ice cover (*P* > 0.05).

### 3.3. Variation between Wetlands by Year

Average species richness and average species diversity were not significantly different during either year (Table 1). Both average species richness and diversity values for PC2013 increased slightly in the second winter. There was a 42.4% overlap in species composition between the 2 wetlands during 2013-2014 and a 28.7% overlap during 2014-2015. During the first winter period, PC2001 had a significantly higher percent composition of Bufflehead (PC2001 = 10.92%, PC2013 = 0.0%, *G*_1_ = 15.1, *P* < 0.001), Gadwall (PC2001 = 10.52%, PC2013 = 0.0%, *G*_1_ = 14.6, *P* < 0.001), Lesser Scaup (PC2001 = 10.41%, PC2013 = 0.0%, *G*_1_ = 14.4, *P* < 0.001), and Ring-Necked Duck (PC2001 = 17.90%, PC2013 = 0.0%, *G*_1_ = 24.8, *P* < 0.001), while PC2013 had a greater percent composition of* Lophodytes cucullatus* L. (Hooded Merganser; PC2001 = 2.74%, PC2013 = 25.61%, *G*_1_ = 21.3, *P* < 0.001) and Wood Duck (PC2001 = 2.06%, PC2013 = 20.73%, *G*_1_ = 17.8, *P* < 0.001). The percent composition of the other 16 waterbird species was similar between the 2 wetlands (*P* > 0.05). During the second winter, PC2001 again had significantly higher percent compositions of Bufflehead (PC2001 = 10.47%, PC2013 = 1.0%, *G*_1_ = 9.1, *P* < 0.005) and Ring-Necked Duck (PC2001 = 16.96%, PC2013 = 0.0%, *G*_1_ = 23.5, *P* < 0.001), along with Canada Goose (PC2001 = 33.77%, PC2013 = 13.0%, *G*_1_ = 9.6, *P* < 0.005). Meanwhile, PC2013 had significantly higher percent compositions of* Anas rubripes* (Brewster) (American Black Duck; PC2001 = 1.30%, PC2013 = 12.50%, *G*_1_ = 10.5, *P* < 0.005), Mallard (PC2001 = 6.72%, PC2013 = 27.0%, *G*_1_ = 13.1, *P* < 0.001), and Wood Duck (PC2001 = 1.60%, PC2013 = 38.50%, *G*_1_ = 42.1, *P* < 0.001). The other 16 waterbird species were similar in composition between the wetlands (*P* > 0.05).

There was no significant difference in average total species use between the 2 wetlands in either year (Table 1). Average total species use appeared to increase at PC2013 from the first winter to the second. Of the 23 species observed in 2013-2014, there were no significant differences in the average species use. The proportion of surveys in which no waterbirds were observed was greater at PC2013 (PC2001: 0.18, PC2013: 0.63, *P* < 0.05) during the first winter, but not during the second. There was no difference in average percent ice cover at the 2 wetlands during either year (*P* > 0.05).

### 3.4. Variation between Wetlands by Month

Species richness was originally found to be significantly greater at PC2001 in November 2013 (PC2001: 5.50 ± 0.87, PC2013: 0.25 ± 0.25, *F*_1,6_ = 33.92, *P* = 0.001), March 2014 (PC2001: 11.0 ± 1.41, PC2013: 2.60 ± 0.51, *F*_1,8_ = 31.22, *P* < 0.001), and March 2015 (PC2001: 11.40 ± 0.40, PC2013: 3.20 ± 0.58, *F*_1,8_ = 134.5, *P* < 0.001). However, those results were found to be insignificant when species richness was quantified as the average number of species per ha per week, which compensates for differences in the 2 wetlands' areas. The difference in species richness was insignificant in all other months (*P* > 0.05). Species diversity followed a similar trend. Diversity was higher at PC2001 in November 2013 (PC2001: 1.173 ± 0.16, PC2013: 0.0 ± 0.0, *F*_1,6_ = 54.78, *P* < 0.001) and March 2015 (PC2001: 1.775 ± 0.04, PC2013: 0.942 ± 0.0, *F*_1,8_ = 25.49, *P* = 0.001). Diversity values of both wetlands in all other months were not significant (*P* > 0.05). The proportion of surveys during which no waterbirds were observed tended to be greater for PC2013 than for PC2001. Average percent ice cover similarly tended to be greater at PC2013, though there was a differences of less than 13% between the 2 wetlands in all months. Ice cover and the proportion of surveys without waterbirds were correlated at PC2001 (*R*^2^ = 0.71, *P* = 0.002) but not at PC2013 (*R*^2^ = 0.22, *P* = 0.17).

Species composition varied between wetlands and among months (Tables 2 and 3). PC2001 tended to have significantly higher percent compositions of* Fulica americana* (Gmelin) (American Coot), Bufflehead, Canada Goose, and Gadwall, while PC2013 tended to have a higher percent composition of Hooded Merganser and Wood Duck. Individual species use did not differ in any month during the first winter (*P* > 0.05), but total species use in March 2014 was greater at PC2001 (PC2001: 10.19 ± 1.10, PC2013: 1.93 ± 0.39, *F*_1,8_ = 50.29, *P* = 0.0001) (see Supplementary Data Table in Supplementary Material available online at https://doi.org/10.1155/2017/1730130). In addition, the number of observed species was almost four times higher at PC2001 (*n* = 19; for PC2013, *n* = 5) in March 2014. In the second winter, there were no differences in average individual or total species use from November 2014 to February 2015 (*P* > 0.05). In March 2015, total species use was not significantly different, but use by Bufflehead (PC2001: 1.09 ± 0.02, PC2013: 0.0 ± 0.0, *F*_1,8_ = 29.22, *P* = 0.0006), Canada Goose (PC2001: 3.50 ± 0.72, PC2013: 0.667 ± 0.0, *F*_1,8_ = 15.62, *P* = 0.004), and Lesser Scaup (PC2001: 0.64 ± 0.13, PC2013: 0.0 ± 0.0, *F*_1,8_ = 23.19, *P* = 0.001) was higher at PC2001.

### 3.5. Trends in Waterbird Use

**Figure 3:**
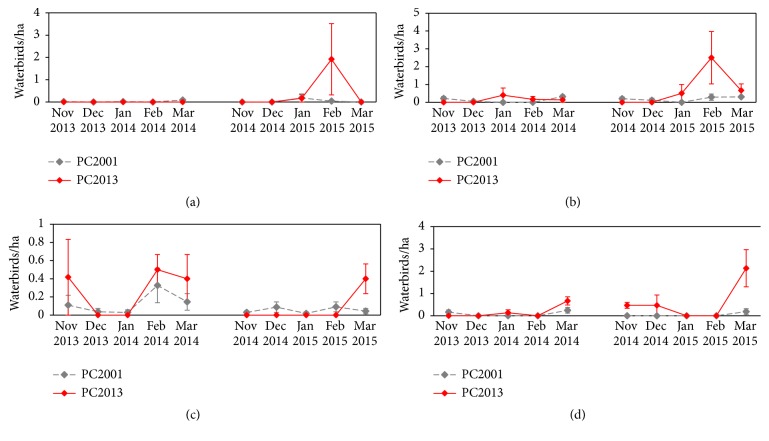
Average American Black Duck (a), Mallard (b), Hooded Merganser (c), and Wood Duck (d) use per ha at PC2001 and PC2013 from November to March 2013-2014 and 2014-2015.

Eleven waterbird species (American Black Duck, American Coot, Bufflehead, Canada Goose, Gadwall, Hooded Merganser, Lesser Scaup, Mallard, Pied-Billed Grebe, Ring-Necked Duck, and Wood Duck) comprised at least 2.0% at either wetland during the 2 years of surveys. Each species differed slightly in patterns of monthly use, which was calculated by averaging the weekly high counts. American Black Ducks, Mallards, Hooded Merganser, and Wood Ducks tended to have higher use at PC2013 (Figure 3). American Black Ducks and Mallards had similar use curves, with a distinct peak in use of PC2013 during February 2015. Hooded Mergansers used the wetlands differently in the first and second years of the study. During the first winter, use was highest in PC2013 in November 2013, February 2014, and March 2014, but in the second year, use was high only in March 2015. In both years, Wood Ducks had the highest use in both wetlands in March.

**Figure 4:**
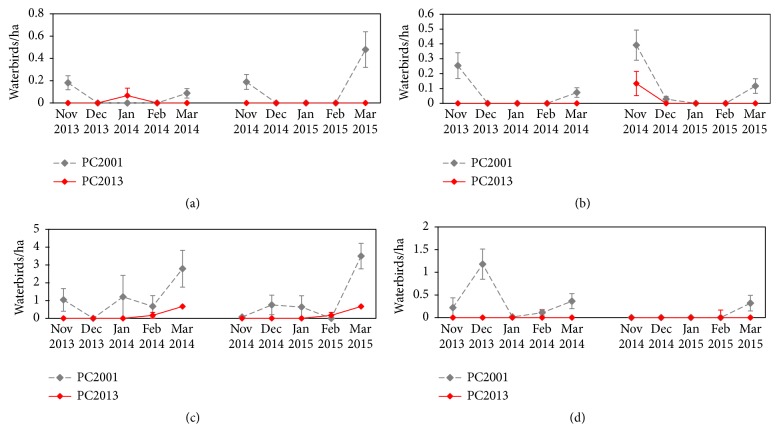
Average American Coot (a), Pied-Billed Grebe (b), Canada Goose (c), and Gadwall (d) use per ha at PC2001 and PC2013 from November to March 2013-2014 and 2014-2015.

**Figure 5:**
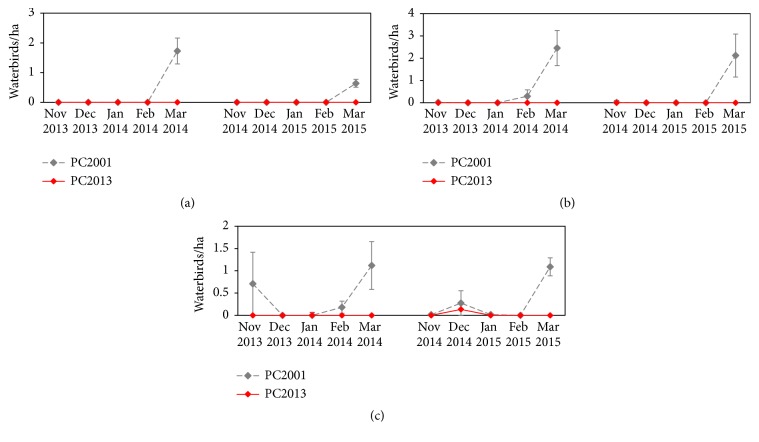
Average diving duck (Lesser Scaup (a), Ring-Necked Duck (b), and Bufflehead (c)) use per ha at PC2001 and PC2013 from November to March 2013-2014 and 2014-2015.

American Coot and Pied-Billed Grebes had higher uses at PC2001 in November and March of both years (Figure 4). Canada Geese and Gadwall tended to increase use in March. During the first winter, Gadwall use peaked in December 2013, but that trend was not repeated the second winter. Lesser Scaup and Ring-Necked Duck had similar use curves (Figure 5). Few to no ducks were detected until March, when use was highest both years. Similarly, Bufflehead use was highest in March of both years, though they also used PC2001 in November 2013 and both wetlands in December 2014.

## 4. Discussion

### 4.1. Waterbird Diversity and Abundance

To our knowledge, this was the first study to evaluate winter waterbird use of differently aged created wetlands in the Central Appalachians. Over the two 5-month periods during our study, we observed the wetlands harboring 3,340 waterbirds belonging to 27 species. They provided food and habitat to a diversity of migratory and wintering waterbirds, which in turn contributed to regional biodiversity and recreational hunting opportunities. At the wetland scale, winter waterbird species richness was not significantly different when wetland area was compensated for. Similarly, average species richness was not significantly different between the 2 wetlands in individual years, though both average species richness and diversity values for PC2013 increased slightly in the second winter, which may indicate that those metrics will increase over time. The greatest disparities in species richness and diversity tended to occur in November 2013, March 2013, and March 2014, while the least disparities occurred in January and February 2013 and 2014. These trends indicate that PC2013 may not attract as many waterbird species during migration (November and March) as PC2001.

The overlap in species composition ranged from 0% in January 2014 to 63% in February 2015, with an average of 25% across months. Differences in species composition were greatest from November to December 2013 and least in January and February 2015. Percent overlap increased or stayed similar in months from the first winter to the second winter. PC2001 generally had higher percent composition of American Coot, Bufflehead, Canada Goose, Gadwall, Lesser Scaup, and Ring-Necked Duck.

Certain waterbirds, including Buffleheads, Canada Geese, Gadwall, Lesser Scaup, and Ring-Necked Duck, tended to have higher use at PC2001. Differences in use were highest in March 2015. Average total species use tended to be higher at PC2001, though there was generally no statistical difference between the wetlands. The difference in total species use was less distinct in the second winter, which may indicate increased habitat availability or quality at PC2013 as the recently created wetland developed and matured. Because waterfowl exhibit site philopatry in the winter and are known to explore new sites, it is also possible that individuals that wintered at PC2013 the first year would come back again the next year and that additional individuals would discover the new wetland, increasing overall use and abundance [32].

Trends in waterbird use were variable. For many waterbirds, particularly diving ducks, use was highest in March during migration. With some exceptions, mid-winter waterbird use was limited. Based on the species richness, diversity, composition, and use results, it appears that the ability of PC2013 is similar to PC2001 in providing habitat for wintering waterbirds but not migrating waterbirds. Furthermore, PC2013 provides different habitat types that appear to favor a different winter waterbird community than PC2001, as its community composition tends to comprise Hooded Mergansers, Mallards, and Wood Ducks. For instance, American Black Ducks, Mallards, and Wood Ducks were observed using the stream within the PC2013 wetland complex when the wetland itself was covered in ice, and Wood Ducks were often found in the upper portion of the wetland, which is interspersed with trees and shrubs.

Certain disparities in species diversity, composition, and use might be explained by differences in wetland area, water depth, microtopography, and vegetative cover. Wetland size can predict waterbird richness and species abundance [33, 34]. We compensated for wetland size differences by dividing species richness and use by the area of the respective wetland. However, larger wetlands tend to have more variable spatial configurations and higher habitat heterogeneity [35]. Concomitantly, these larger wetlands can support a greater diversity of waterbirds with different habitat preferences [1, 9, 36, 37]. For instance, waterbird species that forage in open or deepwater habitats are restricted to relatively large wetlands or ponds [35]. Small wetlands are generally associated with lower species diversity [33]. In our study, both wetlands were relatively similar in habitat on a landscape scale. At a smaller spatial scale, it is possible that the larger PC2001 contained more microhabitat variability. Furthermore, PC2001 had a higher percentage of core habitat (39.2%) than PC2013 (5.3%), due to its size and configuration. With possibly higher microhabitat diversity and more core area, it is reasonable that the larger PC2001 experienced greater species use than PC2013.

Water depth and microtopography also play a role in shaping waterbird communities. Many studies cite water depth as an important variable that affects waterbird use of wetland habitats [9, 38, 39]. Water depth has an impact on waterbirds due to their morphology and feeding habits [40, 41]. Wading and dabbling waterbirds generally require shallow water to forage, and water depth limits their access to foraging habitat [41]. Small shorebirds use water depths of <5 cm, large shorebirds use 5–11 cm, and large dabbling ducks use >20 cm [39]. Diving waterbirds require a minimum depth of >25 cm and can forage in water that is several meters deep [41]. The numbers of waterbird, dabbling duck, and wading bird species tend to increase in shallow wetlands, while the number of diving duck species increases in deeper wetlands [9]. In corroboration with our results, Colwell and Taft [9] found that Gadwall and American Coot tended to occur at higher densities in deep wetlands. In addition, diving ducks such as Bufflehead, Lesser Scaup, and Ring-Necked Duck had higher percent species composition and use at PC2001, which was deeper than PC2013.

Though we were unable to directly discern the effects of age on differences between the 2 wetlands, we can comment on possible indirect effects. As a wetland ages and matures, habitat availability or quality may increase. Wetland vegetation, another major factor that influences waterbird use of wetlands, can become established and flourish over time. Vegetation and habitat heterogeneity are related [42]. Froneman et al. [36] found that structural diversity of vegetation in and around farm ponds was important in determining usage by waterbirds. However, too dense vegetative cover can decrease waterbird use [40]. Vegetation tends to be scarce in winter, the time period of our study, but seeds are an important food resource, and trees are used by species such as* Ardea herodias* L. (Great Blue Herons) and Belted Kingfishers. Craig and Beal [42] posit that marshes of the same size with similar habitat conditions should attract similar species of birds. Thus, the differences in species composition at PC2001 and PC2013 are likely due to varying water depths and divergent habitat variables, possibly mediated by age.

### 4.2. Related Studies

The results of our study were somewhat similar to other previously conducted in the region. Balcombe et al. [19] evaluated breeding avian and anuran communities in 11 mitigation and 4 reference wetlands throughout West Virginia. They found that Wood Ducks were more abundant in mitigation wetlands, while the density of Great Blue Herons was similar between wetland types. In addition, they found that waterbird and waterfowl abundance were higher in mitigation wetlands than reference wetlands. Balcombe et al. [20] attempted to determine if mitigation wetlands in West Virginia were adequately supporting ecological communities relative to naturally occurring reference wetlands and to attribute specific characteristics in wetland habitat with trends in wildlife abundance across wetlands. They found that abundance of waterbirds at mitigated wetlands was affected by age, benthic invertebrate diversity, percent emergent vegetation, percent open water, size, and vegetation diversity. Furthermore, Balcombe et al. [20] ranked mitigation wetlands consistently higher than reference wetlands.

However, studies evaluating created versus natural wetland function vary in their results. Outside of the Central Appalachian region, another study investigated avian communities in created and natural wetlands in Virginia. Desrochers et al. [24] tested the hypothesis that created wetlands provide avian habitat lost via wetland destruction by comparing breeding and wintering birds on 11 small created salt marshes with those on 11 natural reference salt marshes. They found that created salt marshes had lower avian abundance and richness than reference salt marshes during the breeding season. However, observed bird use outside of the breeding season did not differ. Desrochers et al. [24] concluded that the created wetlands they surveyed failed to completely replicate the bird communities observed on nearby natural reference salt marshes. Another study in Virginia assessed ecological conditions in a created tidal marsh and 2 natural reference tidal marshes. Havens et al. [43] found that bird species richness and diversity were similar among the created and natural marshes, and wading birds appeared to show a significant preference for the created marsh. Further to the south, White and Main [29] studied waterbird use of created wetlands in golf-course landscapes in Florida. They found that created golf-course ponds were capable of attracting various species of waterbirds. However, they suggested that the value of golf-course ponds may be enhanced through modifications to the vegetation and hydrology designed to appeal to specific waterbird guilds. In New York, Brown and Smith [44] looked at breeding season bird use of recently restored and natural wetlands. They compared the relative abundance and density of birds using 18 restored wetlands and 8 natural wetlands. Abundances of species did not differ between restored and natural wetlands in any year, but densities were consistently lower at restored sites and bird communities were significantly less similar between restored and natural sites than among restored sites. Brown and Smith [44] conclude that the restoration program successfully increased the amount of bird habitat available in the region, but the restored wetland sites did not entirely replace the habitat functions of natural wetlands during their study. Finally, though they did not specifically survey for waterbirds, Confer and Niering [45] assessed wildlife in created and natural wetlands in Connecticut and observed higher wildlife activity in the natural wetlands.

Similar to the findings of White and Main [29], the 2 created wetlands in our study proved capable of attracting many species of waterbirds. Balcombe et al. [19] indicated that mitigated wetlands in West Virginia were able to support various wildlife species. It is possible that PC2013 will increase in its ability to provide habitat for waterbirds as time progresses. Native wetland plant species diversity and richness have been found to increase with wetland age, which may increase the attractiveness of habitat to waterbirds [46]. Furthermore, average total species use, richness, and diversity of waterbirds using PC2013 seemed to increase from the first winter to the next. Though the community compositions of PC2013 and PC2001 are unlikely to ever be identical due to variation in certain habitat features, species use of PC2013 may continue to increase in coming years.

### 4.3. Management Implications

Our analyses were designed to quantify the differences and changes in the waterbird communities of 2 wetlands of different ages during 2 winter seasons. Though the results of this study are limited in direct applications to the development and management of PC2013, they provide insight into the potential impacts of newly created wetland habitat on local and migrant waterbird species in the Central Appalachians during the nonbreeding season. Our results further highlight important factors of wetland construction that must be taken into account. When designing and creating wetlands, it is important to consider management objectives, wetland size, water depth, topography, and vegetation. Wetland size and water depth influence habitat diversity and waterbird use [9, 36]. To maximize species richness and diversity of wintering waterbirds, managers should ensure that the water depth of wetlands is an average of 10–20 cm, with a range of depths that will attract a large number of species [9]. Larger wetlands tend to support more waterbirds, but small wetlands that are used seasonally by waterbirds can still be important in maintaining local and regional populations [42]. Finally, structural diversity in vegetation as well as the growth and seed production of beneficial wetlands plants should be promoted to provide cover and food for waterbirds.

## Supplementary Material

The Supplementary Data Table contains a summary of mean monthly waterbird species use at the two wetlands (PC2001 and PC2013) in Pleasant Creek WMA, WV, from November to March 2013–2014 and 2014–2015. Individual and total species use were calculated using weekly high species counts per hectare.

## Figures and Tables

**Table 1:** Summary of overall and annual waterbird species use, richness, and diversity with their means and standard errors at 2 created wetlands (PC2001 and PC2013) in Pleasant Creek WMA, WV, from November to March 2013-2014 and 2014-2015. Individual and total species use was calculated using weekly high species counts per ha, while richness and diversity were calculated using total waterbird counts. Bolded means are significant following the use of the sequential Bonferroni approach. Italicized means indicate a result that was rendered insignificant after compensating for differences in wetland size.

Common name	PC2001		PC2013		*P*	2013-14 PC2001		2013-14 PC2013		*P*	2014-15 PC2001		2014-15 PC2013		*P*
	Mean	SE	Mean	SE		Mean	SE	Mean	SE		Mean	SE	Mean	SE	
American Black Duck	0.032	0.02	0.185	0.15	0.313	0.026	0.01	0.000	0.00	0.066	0.038	0.03	0.362	0.29	0.274
American Coot	0.100	0.03	0.007	0.01	0.003	0.053	0.02	0.015	0.02	0.146	0.145	0.05	0.000	0.00	0.009
American Wigeon	0.019	0.01	0.000	0.00	0.156	0.026	0.03	0.000	0.00	0.323	0.013	0.01	0.000	0.00	0.155
Belted Kingfisher	0.031	0.01	0.022	0.01	0.555	0.023	0.01	0.015	0.02	0.653	0.038	0.01	0.029	0.02	0.694
Bufflehead	**0.358**	**0.11**	**0.015**	**0.01**	**0.002**	0.416	0.19	0.000	0.00	0.034	0.303	0.11	0.029	0.03	0.024
Blue-Winged Teal	0.016	0.01	0.000	0.00	0.055	0.023	0.01	0.000	0.00	0.102	0.009	0.01	0.000	0.00	0.323
Cackling Goose	0.005	0.00	0.000	0.00	0.179	0.003	0.00	0.000	0.00	0.323	0.006	0.01	0.000	0.00	0.323
Canada Goose	**1.132**	**0.27**	**0.178**	**0.04**	**0.001**	1.217	0.41	0.182	0.06	0.018	1.051	0.35	0.174	0.06	0.017
Canvasback	0.048	0.02	0.000	0.00	0.010	0.056	0.02	0.000	0.00	0.021	0.041	0.03	0.000	0.00	0.156
Common Merganser	0.006	0.00	0.007	0.01	0.912	—	—	—	—	—	0.013	0.01	0.014	0.01	0.912
Gadwall	**0.211**	**0.06**	**0.000**	**0.00**	**0.001**	0.360	0.12	0.000	0.00	0.003	0.069	0.04	0.000	0.00	0.125
Great Egret	0.003	0.00	0.000	0.00	0.320	—	—	—	—	—	0.006	0.01	0.000	0.00	0.323
Greater Scaup	0.034	0.02	0.000	0.00	0.067	0.036	0.03	0.000	0.00	0.235	0.032	0.02	0.000	0.00	0.155
Great Blue Heron	0.045	0.01	0.030	0.01	0.426	0.053	0.02	0.030	0.02	0.484	0.038	0.01	0.029	0.02	0.705
Green-Winged Teal	0.019	0.01	0.000	0.00	0.106	—	—	—	—	—	0.038	0.02	0.000	0.00	0.103
Horned Grebe	0.002	0.00	0.000	0.00	0.320	0.003	0.00	0.000	0.00	0.323	—	—	—	—	—
Hooded Merganser	0.089	0.02	0.170	0.06	0.191	0.125	0.05	0.258	0.10	0.249	0.054	0.02	0.087	0.05	0.516
Lesser Scaup	0.263	0.10	0.000	0.00	0.007	0.393	0.18	0.000	0.00	0.037	0.139	0.06	0.000	0.00	0.030
Lesser Yellowlegs	0.005	0.00	0.000	0.00	0.320	—	—	—	—	—	0.009	0.01	0.000	0.00	0.323
Mallard	0.155	0.03	0.415	0.17	0.135	0.122	0.04	0.152	0.10	0.781	0.186	0.05	0.667	0.31	0.136
Northern Pintail	0.010	0.01	0.000	0.00	0.179	0.020	0.01	0.000	0.00	0.179	—	—	—	—	—
Pied-Billed Grebe	0.090	0.02	0.015	0.01	0.005	0.063	0.03	0.000	0.00	0.020	0.117	0.04	0.030	0.02	0.074
Redhead	0.008	0.01	0.000	0.00	0.226	0.013	0.01	0.000	0.00	0.323	0.003	0.00	0.000	0.00	0.323
Ring-Necked Duck	0.537	0.19	0.000	0.00	0.006	0.614	0.28	0.000	0.00	0.033	0.464	0.27	0.000	0.00	0.089
Ruddy Duck	0.007	0.00	0.000	0.00	0.041	0.010	0.01	0.000	0.00	0.076	0.003	0.00	0.000	0.00	0.323
Tundra Swan	0.015	0.01	0.000	0.00	0.208	0.030	0.02	0.000	0.00	0.208	—	—	—	—	—
Wood Duck	0.063	0.02	0.430	0.14	0.011	0.086	0.04	0.182	0.08	0.261	0.041	0.03	0.667	0.26	0.020
Total species use	**3.283**	**0.60**	**1.030**	**0.24**	**0.001**	3.770	0.90	0.833	0.19	0.003	2.818	0.81	2.087	0.65	0.483
Species richness	***10.1***	***1.68***	***3.2***	***0.47***	***0.001***	10.8	2.48	2.8	0.66	0.014	9.4	2.50	3.6	0.68	0.056
Species diversity	1.441	0.18	0.831	0.13	0.013	1.448	0.29	0.771	0.22	0.10	1.435	0.25	0.890	0.15	0.099

**Table 2:** Summary of significant differences in monthly waterbird species percent composition at 2 created wetlands (PC2001 and PC2013) in Pleasant Creek WMA, WV, from November to March 2013-2014. All of the following results are significant following the use of the sequential Bonferroni approach.

Month	% composition overlap	Species	PC2001	PC2013	*G*_1_	*P*
November 2013	2.7	American Coot	7.59	0.00	10.5	<0.005
		Bufflehead	17.41	0.00	24.1	<0.001
		Canada Goose	39.73	0.00	55.1	<0.001
		Gadwall	8.48	0.00	11.8	<0.001
		Hooded Merganser	2.68	100.0	117.5	<0.001
		Mallard	6.70	0.00	9.3	<0.005
		Pied-Billed Grebe	8.93	0.00	12.4	<0.001

December 2013	8.3	Belted Kingfisher	3.18	50.0	49.6	<0.001
		Gadwall	76.43	0.00	106.0	<0.001
		Great Blue Heron	5.10	50.0	42.4	<0.001
		Ruddy Duck	3.18	0.00	11.5	<0.001
		Tundra Swan	8.28	0.00	11.5	<0.001

January 2014	0.0	American Coot	0.00	11.11	15.4	<0.001
		Canada Goose	87.37	0.00	121.1	<0.001
		Mallard	0.00	66.67	92.4	<0.001
		Wood Duck	0.00	22.22	30.8	<0.001

February 2014	54.5	Bufflehead	12.20	0.00	16.9	<0.001
		Hooded Merganser	21.14	50.0	12.1	<0.001
		Mallard	0.00	16.67	23.1	<0.001
		Ring-Necked Duck	13.01	0.00	18.0	<0.001

March 2014	35.4	Bufflehead	11.91	0.00	16.5	<0.001
		Hooded Merganser	1.04	18.52	19.0	<0.001
		Lesser Scaup	15.83	0.00	21.9	<0.001
		Ring-Necked Duck	25.74	0.00	35.7	<0.001
		Wood Duck	2.00	27.78	26.6	<0.001

**Table 3:** Summary of significant differences in monthly waterbird species percent composition at 2 created wetlands (PC2001 and PC2013) in Pleasant Creek WMA, WV, from November to March 2014-2015. All of the following results are significant following the use of the sequential Bonferroni approach.

Month	% composition overlap	Species	PC2001	PC2013	*G*_1_	*P*
November 2014	20.8	American Coot	16.67	0.00	23.1	<0.001
		Belted Kingfisher	0.83	13.33	13.3	<0.001
		Mallard	11.67	0.00	16.2	<0.001
		Pied-Billed Grebe	45.0	13.33	18.2	<0.001
		Wood Duck	0.00	60.00	83.2	<0.001

December 2014	12.8	Canada Goose	57.05	0.00	79.1	<0.001
		Mallard	11.54	0.00	16.0	<0.001
		Wood Duck	0.00	83.33	115.5	<0.001

January 2015	25.0	Canada Goose	66.04	0.00	91.6	<0.001
		Mallard	75.00	0.00	104.0	<0.001

February 2015	63.0	American Black Duck	7.69	41.07	25.1	<0.001
		Hooded Merganser	19.23	0.00	26.7	<0.001

March 2015	29.0	American Coot	6.50	0.00	9.0	<0.005
		Bufflehead	12.16	0.00	16.9	<0.001
		Lesser Scaup	7.34	0.00	10.2	<0.005
		Ring-Necked Duck	23.17	0.00	32.1	<0.001
		Wood Duck	2.20	53.20	58.3	<0.001
