# Supplementary material for: Winter Waterbird Community Composition and Use at Created Wetlands in West Virginia, USA
Source: Scientifica (Cairo). 2017 Mar 12;2017:1730130. doi: 10.1155/2017/1730130 (PMC5366207; doi:10.1155/2017/1730130)
Supplement: Supplementary file 1 — The Supplementary Data Table contains a summary of mean monthly waterbird species use at the two wetlands (PC2001 and PC2013) in Pleasant Creek WMA, WV, from November to March 2013–2014 and 2014–2015. Individual and total species use were calculated using weekly high species counts per hectare. [file 1730130.f1.pdf]

Supplementary Data Table: Summary of monthly waterbird species use with means and standard errors at the two wetlands (PC2001 and PC2013) in Pleasant Creek WMA, WV, from November to March 2013–2014 and 2014–2015. Individual and total species use was calculated using weekly high species counts per hectare. Bolded means are significant following the use of the sequential Bonferroni approach.

**Common Name**	**Nov 2013 PC2001**		**Nov 2013 PC2013**			**Dec 2013 PC2001**		**Dec 2013 PC2013**			**Jan 2014 PC2001**		**Jan 2014 PC2013**		
	**Mean**	**SE**	**Mean**	**SE**	***P***	**Mean**	**SE**	**Mean**	**SE**	***P***	**Mean**	**SE**	**Mean**	**SE**	***P***
American Black Duck	0.018	0.02	0.000	0.00	0.356	---	---	---	---	---	0.015	0.01	0.000	0.00	0.347
American Coot	0.181	0.06	0.000	0.00	0.028	---	---	---	---	---	0.000	0.00	0.067	0.07	0.347
American Wigeon	---	---	---	---	---	---	---	---	---	---	---	---	---	---	---
Belted Kingfisher	0.018	0.02	0.000	0.00	0.356	0.054	0.03	0.083	0.08	0.760	0.044	0.02	0.000	0.00	0.040
Bufflehead	0.708	0.71	0.000	0.00	0.356	---	---	---	---	---	---	---	---	---	---
Blue-winged Teal	---	---	---	---	---	0.018	0.02	0.000	0.00	0.356	---	---	---	---	---
Cackling Goose	---	---	---	---	---	---	---	---	---	---	0.015	0.01	0.000	0.00	0.347
Canada Goose	1.034	0.64	0.000	0.00	0.156	---	---	---	---	---	1.205	1.20	0.000	0.00	0.347
Canvasback	---	---	---	---	---	---	---	---	---	---	---	---	---	---	---
Common Merganser	---	---	---	---	---	---	---	---	---	---	---	---	---	---	---
Gadwall	0.218	0.22	0.000	0.00	0.356	1.179	0.33	0.000	0.00	0.012	0.015	0.01	0.000	0.00	0.347
Great Egret	---	---	---	---	---	---	---	---	---	---	---	---	---	---	---
Greater Scaup	---	---	---	---	---	---	---	---	---	---	---	---	---	---	---
Great Blue Heron	0.018	0.02	0.000	0.00	0.356	0.054	0.02	0.083	0.08	0.746	0.044	0.03	0.000	0.00	0.172
Green-winged Teal	---	---	---	---	---	---	---	---	---	---	---	---	---	---	---
Horned Grebe	---	---	---	---	---	---	---	---	---	---	---	---	---	---	---
Hooded Merganser	0.109	0.11	0.417	0.42	0.502	0.036	0.04	0.000	0.00	0.356	0.029	0.03	0.000	0.00	0.347
Lesser Scaup	---	---	---	---	---	---	---	---	---	---	---	---	---	---	---
Lesser Yellowlegs	---	---	---	---	---	---	---	---	---	---	---	---	---	---	---
Mallard	0.218	0.11	0.000	0.00	0.088	0.054	0.05	0.000	0.00	0.356	0.000	0.00	0.400	0.40	0.347
Northern Pintail	---	---	---	---	---	---	---	---	---	---	---	---	---	---	---
Pied-billed Grebe	0.254	0.09	0.000	0.00	0.026	---	---	---	---	---	---	---	---	---	---
Redhead	---	---	---	---	---	---	---	---	---	---	---	---	---	---	---
Ring-necked Duck	0.018	0.02	0.000	0.00	0.356	---	---	---	---	---	---	---	---	---	---
Ruddy Duck	0.018	0.02	0.000	0.00	0.356	0.036	0.02	0.000	0.00	0.134	---	---	---	---	---
Tundra Swan	0.018	0.02	0.000	0.00	0.356	0.145	0.12	0.000	0.00	0.280	---	---	---	---	---
Wood Duck	0.163	0.10	0.000	0.00	0.138	---	---	---	---	---	0.000	0.00	0.133	0.13	0.347
Total species use	2.994	1.23	0.417	0.42	0.095	1.579	0.37	0.167	0.10	0.010	1.364	1.24	0.600	0.37	0.571

Supplementary Data Table. Continued.

Common Name	Feb 2014 PC2001		Feb 2014 PC2013			Mar 2014 PC2001		Mar 2014 PC2013			Nov 2014 PC2001		Nov 2014 PC2013		
	**Mean**	**SE**	**Mean**	**SE**	***P***	**Mean**	**SE**	**Mean**	**SE**	***P***	**Mean**	**SE**	**Mean**	**SE**	***P***
American Black Duck	---	---	---	---	---	0.087	0.05	0.000	0.00	0.141	---	---	---	---	---
American Coot	---	---	---	---	---	0.087	0.04	0.000	0.00	0.074	0.189	0.07	0.000	0.00	0.023
American Wigeon	---	---	---	---	---	0.1161	0.12	0.000	0.00	0.347	---	---	---	---	---
Belted Kingfisher	---	---	---	---	---	---	---	---	---	---	0.015	0.01	0.067	0.07	0.467
Bufflehead	0.181	0.14	0.000	0.00	0.235	1.118	0.54	0.000	0.00	0.071	0.015	0.01	0.000	0.00	0.347
Blue-winged Teal	---	---	---	---	---	0.087	0.05	0.000	0.00	0.141	---	---	---	---	---
Cackling Goose	---	---	---	---	---	---	---	---	---	---	---	---	---	---	---
Canada Goose	0.671	0.60	0.167	0.17	0.449	2.79	1.03	0.667	0.00	0.074	0.073	0.07	0.000	0.00	0.347
Canvasback	0.073	0.07	0.000	0.00	0.356	0.189	0.05	0.000	0.00	0.008	---	---	---	---	---
Common Merganser	---	---	---	---	---	---	---	---	---	---	0.000	0.00	0.067	0.07	0.347
Gadwall	0.109	0.07	0.000	0.00	0.168	0.363	0.17	0.000	0.00	0.060	---	---	---	---	---
Great Egret	---	---	---	---	---	---	---	---	---	---	---	---	---	---	---
Greater Scaup	---	---	---	---	---	0.160	0.13	0.000	0.00	0.243	---	---	---	---	---
Great Blue Heron	---	---	---	---	---	0.131	0.10	0.067	0.07	0.605	0.087	0.01	0.067	0.07	0.772
Green-winged Teal	---	---	---	---	---	---	---	---	---	---	0.087	0.09	0.000	0.00	0.347
Horned Grebe	---	---	---	---	---	0.015	0.01	0.000	0.00	0.347	---	---	---	---	---
Hooded Merganser	0.327	0.19	0.500	0.17	0.519	0.145	0.09	0.400	0.27	0.393	0.029	0.02	0.000	0.00	0.141
Lesser Scaup	---	---	---	---	---	1.727	0.44	0.000	0.00	0.004	---	---	---	---	---
Lesser Yellowlegs	---	---	---	---	---	---	---	---	---	---	0.044	0.04	0.000	0.00	0.347
Mallard	0.000	0.00	0.167	0.17	0.356	0.319	0.09	0.133	0.13	0.287	0.203	0.1	0.000	0.00	0.073
Northern Pintail	0.073	0.07	0.000	0.00	0.356	0.029	0.03	0.000	0.00	0.347	---	---	---	---	---
Pied-billed Grebe	---	---	---	---	---	0.073	0.03	0.000	0.00	0.056	0.392	0.10	0.133	0.08	0.083
Redhead	---	---	---	---	---	0.058	0.06	0.000	0.00	0.347	---	---	---	---	---
Ring-necked Duck	0.290	0.29	0.000	0.00	0.356	2.453	0.79	0.000	0.00	0.015	0.015	0.01	0.000	0.00	0.347
Ruddy Duck	---	---	---	---	---	---	---	---	---	---	---	---	---	---	---
Tundra Swan	---	---	---	---	---	---	---	---	---	---	---	---	---	---	---
Wood Duck	---	---	---	---	---	0.247	0.12	0.667	0.18	0.094	0.000	0.00	0.467	0.13	0.008
Total species use	1.724	1.41	0.833	0.17	0.554	**10.189**	**1.10**	**1.933**	**0.39**	**0.000**	1.147	0.31	0.800	0.13	0.333

Supplementary Data Table. Continued.

	Dec 2014 PC2001		Dec 2014 PC2013			Jan 2015 PC2001		Jan 2015 PC2013			Feb 2015 PC2001		Feb 2015 PC2013		
	**Mean**	**SE**	**Mean**	**SE**	***P***	**Mean**	**SE**	**Mean**	**SE**	***P***	**Mean**	**SE**	**Mean**	**SE**	***P***
American Black Duck	---	---	---	---	---	0.181	0.18	0.167	0.17	0.954	0.036	0.04	1.917	1.6	0.285
American Coot	---	---	---	---	---	---	---	---	---	---	---	---	---	---	---
American Wigeon	---	---	---	---	---	---	---	---	---	---	---	---	---	---	---
Belted Kingfisher	0.087	0.03	0.000	0.00	0.012	0.018	0.02	0.000	0.00	0.356	0.018	0.02	0.083	0.08	0.474
Bufflehead	0.276	0.28	0.133	0.13	0.654	0.018	0.02	0.000	0.00	0.356	---	---	---	---	---
Blue-winged Teal	---	---	---	---	---	---	---	---	---	---	---	---	---	---	---
Cackling Goose	0.029	0.03	0.000	0.00	0.347	---	---	---	---	---	---	---	---	---	---
Canada Goose	0.755	0.55	0.000	0.00	0.207	0.635	0.63	0.000	0.00	0.356	0.000	0.00	0.167	0.17	0.356
Canvasback	---	---	---	---	---	---	---	---	---	---	---	---	---	---	---
Common Merganser	---	---	---	---	---	---	---	---	---	---	---	---	---	---	---
Gadwall	---	---	---	---	---	---	---	---	---	---	---	---	---	---	---
Great Egret	---	---	---	---	---	---	---	---	---	---	---	---	---	---	---
Greater Scaup	---	---	---	---	---	---	---	---	---	---	---	---	---	---	---
Great Blue Heron	0.044	0.02	0.000	0.00	0.040	---	---	---	---	---	---	---	---	---	---
Green-winged Teal	---	---	---	---	---	---	---	---	---	---	---	---	---	---	---
Horned Grebe	---	---	---	---	---	---	---	---	---	---	---	---	---	---	---
Hooded Merganser	0.087	0.06	0.000	0.00	0.172	0.018	0.02	0.000	0.00	0.356	0.091	0.05	0.000	0.00	0.147
Lesser Scaup	---	---	---	---	---	---	---	---	---	---	---	---	---	---	---
Lesser Yellowlegs	---	---	---	---	---	---	---	---	---	---	---	---	---	---	---
Mallard	0.116	0.12	0.000	0.00	0.347	0.000	0.00	0.500	0.50	0.356	0.290	0.18	2.500	1.47	0.186
Northern Pintail	---	---	---	---	---	---	---	---	---	---	---	---	---	---	---
Pied-billed Grebe	0.029	0.02	0.000	0.00	0.141	---	---	---	---	---	---	---	---	---	---
Redhead	---	---	---	---	---	---	---	---	---	---	---	---	---	---	---
Ring-necked Duck	---	---	---	---	---	---	---	---	---	---	---	---	---	---	---
Ruddy Duck	0.015	0.01	0.000	0.00	0.347	---	---	---	---	---	---	---	---	---	---
Tundra Swan	---	---	---	---	---	---	---	---	---	---	---	---	---	---	---
Wood Duck	0.000	0.00	0.467	0.47	0.347	---	---	---	---	---	---	---	---	---	---
Total species use	1.437	0.85	0.600	0.45	0.409	0.871	0.80	0.667	0.67	0.851	0.435	0.27	4.667	2.92	0.199

Supplementary Data Table. Continued.

Common Name	Mar 2015 PC2001		Mar 2015 PC2013		
	**Mean**	**SE**	**Mean**	**SE**	***P***
American Black Duck	---	---	---	---	---
American Coot	0.479	0.16	0.000	0.00	0.017
American Wigeon	0.058	0.04	0.000	0.00	0.141
Belted Kingfisher	0.044	0.02	0.000	0.00	0.040
Bufflehead	**1.089**	**0.20**	**0.000**	**0.00**	**0.001**
Blue-winged Teal	0.044	0.04	0.000	0.00	0.347
Cackling Goose	---	---	---	---	---
Canada Goose	**3.498**	**0.72**	**0.667**	**0.00**	**0.0004**
Canvasback	0.189	0.12	0.000	0.00	0.143
Common Merganser	0.058	0.04	0.000	0.00	0.141
Gadwall	0.319	0.17	0.000	0.00	0.102
Great Egret	0.029	0.03	0.000	0.00	0.347
Greater Scaup	0.145	0.09	0.000	0.00	0.141
Great Blue Heron	0.044	0.04	0.067	0.07	0.779
Green-winged Teal	0.087	0.06	0.000	0.00	0.172
Horned Grebe	---	---	---	---	---
Hooded Merganser	0.044	0.03	0.400	0.16	0.064
Lesser Scaup	**0.639**	**0.13**	**0.000**	**0.00**	**0.001**
Lesser Yellowlegs	---	---	---	---	---
Mallard	0.305	0.03	0.667	0.37	0.352
Northern Pintail	---	---	---	---	---
Pied-billed Grebe	0.116	0.05	0.000	0.00	0.046
Redhead	0.015	0.01	0.000	0.00	0.347
Ring-necked Duck	2.119	0.96	0.000	0.00	0.059
Ruddy Duck	---	---	---	---	---
Tundra Swan	---	---	---	---	---
Wood Duck	0.189	0.13	2.133	0.83	0.050
Total species use	9.507	1.08	3.933	1.18	0.008
